# Extraordinarily Stable Hairpin-Based Biosensors for Rapid Detection of DNA Ligases

**DOI:** 10.3390/bios13090875

**Published:** 2023-09-08

**Authors:** Ziang Wu, Roujuan Kou, Kun Ni, Rui Song, Yuxuan Li, Tianhu Li, Hao Zhang

**Affiliations:** 1 Research and Development Institute of Northwestern Polytechnical University in Shenzhen, Shenzhen 518057, China; 2 School of Physical Sciences, Great Bay University & Great Bay Institute for Advanced Study, Dongguan 523000, China; 3 School of Chemistry and Chemical Engineering, Northwestern Polytechnical University, Xi’an 710129, China; 4 School of Aeronautics, Northwestern Polytechnical University, Xi’an 710072, China

**Keywords:** DNA ligases, extraordinarily stable hairpins, biomarker detection, fluorescence probes, molecular sensing

## Abstract

DNA ligases are essential enzymes involved in DNA replication and repair processes in all organisms. These enzymes seal DNA breaks by catalyzing the formation of phosphodiester bonds between juxtaposed 5′ phosphate and 3′ hydroxyl termini in double-stranded DNA. In addition to their critical roles in maintaining genomic integrity, DNA ligases have been recently identified as diagnostic biomarkers for several types of cancers and recognized as potential drug targets for the treatment of various diseases. Although DNA ligases are significant in basic research and medical applications, developing strategies for efficiently detecting and precisely quantifying these crucial enzymes is still challenging. Here, we report our design and fabrication of a highly sensitive and specific biosensor in which a stable DNA hairpin is utilized to stimulate the generation of fluorescence signals. This probe is verified to be stable under a wide range of experimental conditions and exhibits promising performance in detecting DNA ligases. We anticipate that this hairpin-based biosensor will significantly benefit the development of new targeting strategies and diagnostic tools for certain diseases.

## 1. Introduction

Deoxyribonucleic acid (DNA) ligase is an essential and ubiquitous enzyme involved in DNA replication, repair, and recombination in prokaryotic and eukaryotic cells. This enzyme catalyzes the formation of phosphodiester bonds between the juxtaposed 5′ phosphate end and 3′ hydroxyl end in double-stranded DNA in the presence of ATP [1,2,3,4]. Regardless of their sources, DNA ligase-catalyzed reactions’ ligation mechanism shares a common feature: the critical step is forming a covalent DNA ligase–adenylate intermediate [5]. In addition, it has been known that DNA ligases in eukaryotes, archaea, and viruses are ATP-dependent, while those in bacteria are NAD-dependent [6,7,8]. As a result, NAD-dependent DNA ligases have been the targets of new antibacterial agents, as they are widely present in bacteria but rare in mammalian cells [9]. DNA ligases have also been used as cancer biomarkers in recent years [10] because their over-expressions and defects are closely correlated with the pathogenesis of cancer and neurodegeneration [11]. Furthermore, DNA ligases have been used as vital tools in in vitro manipulations of DNA, such as DNA nanotechnology, DNA computing, and DNA sensing [12]. Therefore, DNA ligase activity detection is significant for drug development, medical diagnosis, and basic biochemical research. The conventional means for detecting DNA ligases’ activities mainly rely on immunological detection methods, such as Western blot and ELISA, which are complicated and require specialized facilities. In addition, DNA ligase detection through multiple enzymes and multi-step reactions has been developed in the past [13,14]. However, a drawback of these methods is that their detections introduced many unnecessary factors, reducing their specificity and limiting their real-time application.

On the other hand, the concept of an “extraordinarily stable hairpin” was introduced to the research fields of nucleic acid chemistry in 1989 [15,16], which refers commonly to specific short DNA or RNA sequences that possess exceptionally high thermostability and elevated resistance to nucleases. These DNA and RNA sequences usually contain 7 to 10 long nucleotides and display hairpin-like structures. Although the stem sequences of the extraordinarily stable hairpins are relatively short, their melting temperatures (as represented by Hairpin 1 and Hairpin 2 in Table 1) are significantly higher than those of regular DNA hairpins (represented by Hairpin 3 in Table 1). The high thermostability of this extraordinarily stable hairpin has been attributed to the balance between its high bendability and stacking ability in the loop region, favorable formations of B-form DNA in the stem regions, relatively high GC content, and other factors [15,16]. Even though extraordinarily stable hairpins possess unique properties, these readily formed structures have not yet been utilized for detecting DNA modification enzymes. Here, we present T4 DNA ligase as an example, showcasing for the first time our approach to detecting DNA ligase activity through the formation of extraordinarily stable hairpins. Our studies show that these methods are fast, sensitive, and suitable for the real-time detection of DNA ligase activity.

## 2. Materials and Methods

### 2.1. Reagents

Deionized water and Tris-HCl buffer solution (pH 8.0, 1 M) were purchased from Sangon Biotech (Shanghai, China). Lithium chloride was purchased from Shanghai Aladdin Bio-Chem Technology (Shanghai, China). T4 DNA ligase and 10 × T4 DNA ligase reaction buffer (400 mM Tris-HCl, 100 mM MgCl2, 100 mM DTT, 5 mM ATP, pH 7.8) were purchased from Sangon Biotech (Shanghai, China).

### 2.2. Oligonucleotides

All oligonucleotides used in this study were synthesized by Sangon Biotech (Shanghai, China). Nucleotide sequences and the modification details of these oligonucleotides are given in Table 2. Cyanine 3 (Cy3) is a commonly used cyanine fluorescent dye with an excitation peak at 554 nm and an emission peak at 568 nm. Due to its high quantum yield and stability under physiological conditions, Cy3 was used as the fluorophore in our study for labeling the DNA probes. Black Hole Quencher-2 (BHQ-2) is a fluorescence quencher with an effective absorbance range of 550 to 650 nm, which provides low background fluorescence and a better signal-to-noise ratio in our study. BHQ-2 was modified on the DNA probes for effectively quenching the fluorescence signals generated from Cy3 fluorophores.

### 2.3. Fluorescence Spectroscopic Examinations

A 60 μL solution containing DNA probes with or without DNA ligase was placed in a micro quartz cuvette (0.3 cm optical path) at room temperature for fluorescence spectroscopic analysis using F-7100 Fluorescence Spectrophotometer (Hitachi). Fluorescence emission spectra were measured using 515 nm excitation light at an angle 90° and recorded every nanometer from 530 nm to 650 nm. Both excitation and emission slits were set to 5 nm.

### 2.4. Preparation of DNA Probes

A solution containing 900 nM (unless otherwise stated) of Oligo 1, Oligo 2, Oligo 3, and Oligo 4 (Table 2), 40 mM Tris-HCl (pH 8.0), and 20 mM NaCl was mixed to a total volume of 30 µL at room temperature. This solution was then incubated at 95 °C for 5 min then slowly cooled to room temperature.

### 2.5. DNA Ligase-Catalyzed Ligation and Dissociation of DNA Probes

**Figure 1:**
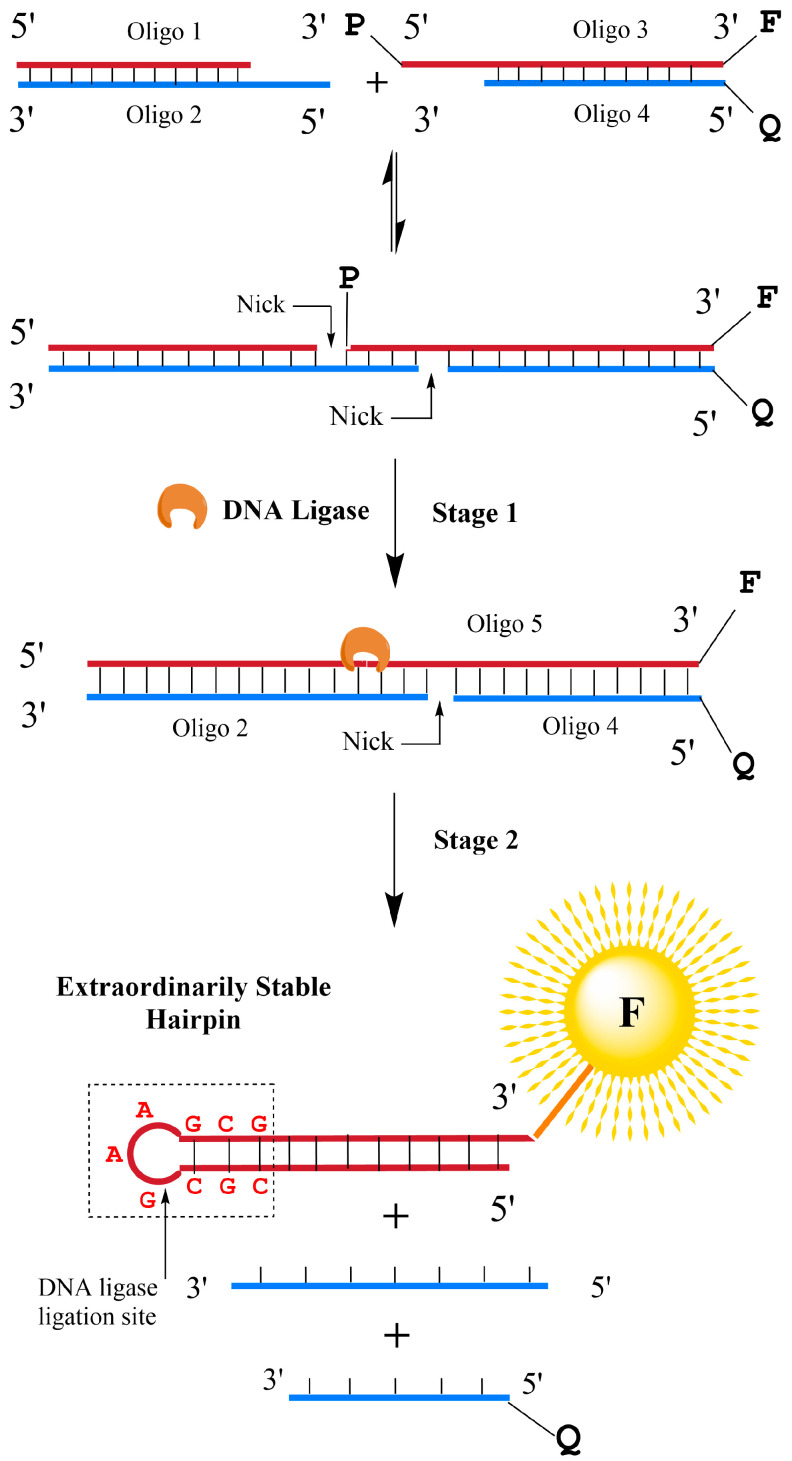
Schematic illustrations of our extraordinarily stable hairpin-based designs for detecting DNA ligase activities. Without DNA ligase, duplex forms of DNAs are in equilibrium, and the fluorophore and quencher moieties always remain in proximity. In the presence of DNA ligase, on the other hand, this enzyme catalyzes the nick-sealing between Oligo 1 and Oligo 3, which leads to the generation of Oligo 5 (Stage 1). Since the newly formed Oligo 5 holds a 5′ CGCGAAGCG 3′ segment in its sequence, it will form an extraordinarily stable hairpin spontaneously (Stage 2). As a result, signals of the previously quenched fluorophore are restored.

As shown in Figure 1, 60 µL solutions containing 40 mM Tris-HCl (pH 7.8), 10 mM NaCl, 10 mM MgCl2, 10 mM DTT, 0.5 mM ATP, 900 nM of DNA probes (unless otherwise stated), and certain concentrations of T4 DNA ligases were prepared and incubated at room temperature for 0.5 h (unless otherwise stated). As shown in Figure 1, the solution was further set at 44 °C for 5 min and then cooled to room temperature over 10 min for efficient dissociation of the fluorophores and quenchers.

## 3. Results and Discussion

### 3.1. Design Strategies of the DNA Probes Targeting DNA Ligases

The unique thermodynamic properties of extraordinarily stable mini-hairpins (ESMHs) [15,16] were fully utilized in our studies to determine the catalytic activity of DNA ligases. Our “one-pot” design strategy for the rapid and efficient detection of DNA ligases is illustrated in Figure 1. By ingeniously designing the chain length, the stable hairpin can preferentially form during annealing, competing with the duplex to achieve the effect of separating the double-stranded DNA. Accordingly, we designed a double-stranded DNA with one end modified with a fluorescent group and a quencher, and the stable hairpin was cleaved to form a sticky end. The DNA design is shown in Table 2.

**Figure 2:**
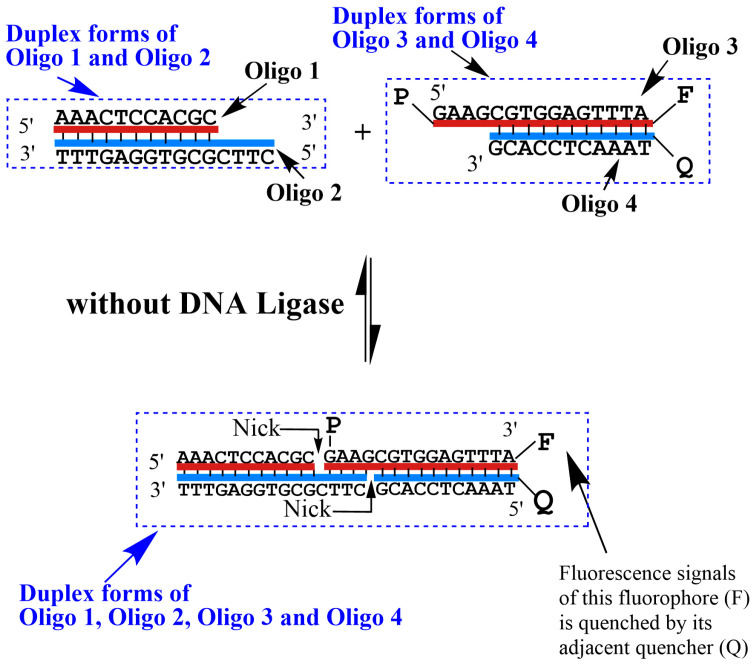
Detailed illustration of the biosensor’s state in the absence of DNA ligase. The probe solution was prepared with 20 mM NaCl in deionized water.

In consideration of the facts that T_m_ (5′ CGCGAAGCG 3′) 88.5 °C > T_m_ (complete duplex) 74 °C > T_m_ (the double-stranded structures formed by Oligo 1 and Oligo 2) 44 °C > T_m_ (the double-stranded structures formed by Oligo 3 and Oligo 4) 38 °C > 37 °C, Oligo 1 and Oligo 3 can be ligated in the presence of DNA ligase to form a long single-stranded Oligo 5, in which an extraordinarily stable hairpin was induced to stimulate the dehybridization of Oligo 5 from the double-stranded structures (Figure 1). The abovementioned T_m_ (melting temperature) values were simulated using the T_m_ Calculator from Thermo Scientific Web Tools, provided by Thermo Scientific. This thermodynamic-driven spontaneous process causes the efficient dissociation of fluorophores and quenchers and produces remarkable fluorescence signals. Without DNA ligases, the double-stranded structures formed by Oligo 3, Oligo 4, and Oligo 5 remain stable upon heating and thus produce no fluorescence signals. The details of our designed biosensors are depicted in Figure 2. They consist of a 26-bp nucleotide sequence with a GC content of 50%. The target structure it aims to form is an extraordinarily stable hairpin, comprising a single-stranded oligonucleotide chain of 26 deoxyribonucleotides. Within this sequence, there are 11 base pairs of complementary pairings. The “nick” refers to the absence of phosphodiester bonds between the adjacent 5′ phosphate and 3′ hydroxyl ends. Between the two “nicks”, there exists a cohesive end featuring four complementary base pairings, which provides sufficient recognition and binding regions for DNA ligases.

### 3.2. Selection of DNA Ligase

The design of our biosensor leverages the DNA ligase’s most universal property, which is its ability to form phosphodiester bonds between the juxtaposed 5′ phosphate end and 3′ hydroxyl end of dsDNA containing cohesive ends in the presence of ATP. Among these ligases, the T4 DNA ligase stands out as the most extensively utilized enzyme in molecular biology and biochemical experiments. Its ubiquity and ease of acquisition, as well as the comprehensive understanding of its properties and activities, facilitate the prediction of its performance under specific conditions. Moreover, the T4 DNA ligase exhibits wide adaptability and high efficiency under varying reaction conditions. Consequently, these factors make the T4 DNA ligase a suitable representative of DNA ligases for our purposes. Additionally, since the detection strategy for DNA ligases primarily relies on the inherent catalytic activity of these enzymes, our current experimental findings can be extrapolated to other DNA ligases as well.

### 3.3. Detection of T4 DNA Ligase Using the DNA Probes

**Figure 3:**
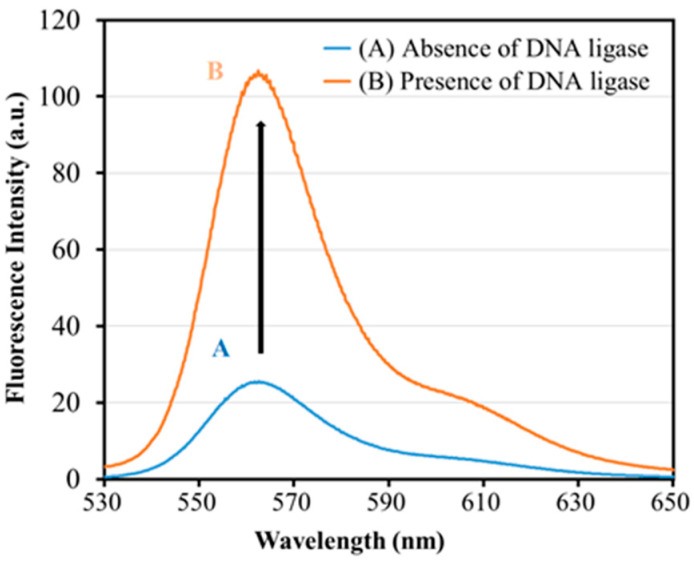
Fluorescence emission spectra in the absence (A) and presence (B) of T4 DNA ligase. DNA probes (900 nM) and T4 DNA ligase (1.3 U/mL) were incubated with 40 mM Tris-HCl (pH 7.8), 10 mM NaCl, 10 mM MgCl2, 10 mM DTT, and 0.5 mM ATP at room temperature for 0.5 h, followed by fluorescence spectroscopic examinations.

A pilot study was carried out first to demonstrate the feasibility of our newly designed DNA probes for detecting the presence of T4 DNA ligase. As depicted in the fluorescence spectroscopic results (Figure 3(A)), the DNA probe exhibited a relatively low fluorescence intensity (the blue curve with a peak value of 24.9 a.u.) in the absence of the T4 DNA ligase, which indicated that only a partial dissociation of the double-stranded structures formed by Oligo 3 and Oligo 4 occurred at room temperature, resulting in a small amount of fluorescence produced by the residual unquenched fluorophore under the excitation light. On the other hand, the fluorescence signal was greatly enhanced (the orange curve with a peak value of 106.7 a.u.) when the DNA probes were incubated with 1.3 U/mL of the T4 DNA ligase (Figure 3(B)). This significant increase in fluorescence intensity indicated that most of Oligo 1 and Oligo 3 were connected by T4 DNA ligase to form Oligo 5. After the dissociation (Stage 2 in Figure 1) process, an extraordinarily stable hairpin was generated from Oligo 5, which resulted in the separation of the Cy3 fluorophore from the BHQ-2 quencher that was modified on the 5′ terminal end of Oligo 4. Consequently, a large amount of fluorescence was induced with the laser excitation at 515 nm, demonstrating our DNA probe’s feasibility in detecting T4 DNA ligase using the F-7100 Fluorescence Spectrophotometer.

### 3.4. Optimization of Experimental Conditions for Effective Detecting of DNA Ligase

**Figure 4:**
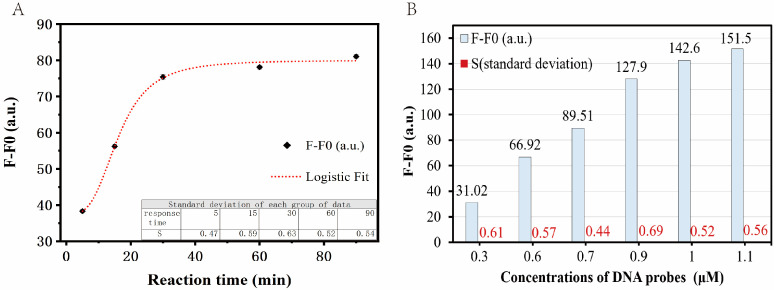
Optimization of experimental conditions for effective detecting of DNA ligase. (**A**) Optimization of the incubation time for the interaction of DNA probes and DNA ligases. Five parallel experiments were performed with a probe concentration of 800 nM and different incubation times of 5, 15, 30, 60, and 90 min to determine the optimal incubation time. All other experimental conditions are described in the Materials and Methods section. (**B**) Optimization of the concentration of DNA probes. To determine the optimal concentration of the DNA probe, six parallel experiments were performed with DNA probe concentrations of 0.3, 0.6, 0.7, 0.9, 1, and 1.1 μM, respectively. All other experimental conditions are described in the Materials and Methods section.

To achieve better sensing performance, several investigations were subsequently carried out to optimize two main factors: incubation time and probe concentration. As shown in Figure 4A, the fluorescence intensity of the DNA probes increases with the incubation time. It reaches a plateau after 30 min (Figure 4A), which suggests that DNA ligases can be rapidly and efficiently detected using our newly developed probes within hours. Therefore, 30 min was chosen as the incubation time for the interaction of DNA probes and DNA ligases in our following experiments. In the ligase-catalyzed DNA ligation process, reaction efficiency is determined by the local equilibrium between four free reactants (Oligos 1–4) and the intermediate formed by these oligonucleotides (DNA duplex with two nick sites), which could be one of the rate-determining steps before the enzyme recognizes and binds to its substrates. In addition, the separation of fluorophores and quenchers is highly dependent on the effective induction of extraordinarily stable hairpins from the nick-containing DNA duplex, which is a thermodynamic competition between different hydrogen bond combinations. Both of the two factors could contribute to the slow rise in fluorescence as shown in Figure 4A.

In addition to the incubation time, the DNA probe concentration was also optimized in our study. In theory, it is evident that fluorescence intensity increases with an increase in probe concentration, particularly in the presence of a relatively high enzyme concentration. As depicted in Figure 4B, under conditions of 1.3 U/mL DNA ligase (a relatively high concentration), we observed that when the concentration of the DNA probes reached 900 nM, the growth rate began to decrease. Further increasing the probe quantity would hardly result in better sensitivities. Thus, 900 nM was selected in our current studies as the standard probe concentration, which can also be adjusted according to the actual situation of the specific application environment.

### 3.5. Measurement of the Limit of Detection (LoD) of DNA Probes

**Figure 5:**
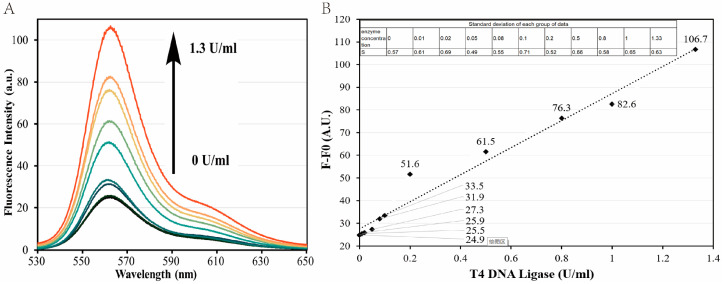
Measurement of the limit of detection (LoD) of DNA probes. (**A**) Fluorescent signal responses with different concentrations of T4 DNA ligases. T4 DNA ligase concentration increases from bottom to top: 0.01, 0.02, 0.05, 0.08, 0.1, 0.2, 0.5, 0.8, 1.0, and 1.3 U/mL. All experimental conditions except for the enzyme concentration were kept the same as in the Materials and Methods section. (**B**) Correlation between fluorescence intensity generated and T4 DNA ligase concentration. The dashed line is obtained by linear regression. T4 DNA ligase unit (U) definitions: in the ATP-PPi exchange reaction, 1 U is the enzyme required to convert 1 nmol [32PPi] to the Norit absorbable form within 20 min at 37 °C.

At optimal conditions, the fluorescence emission spectra of our DNA probes were determined with T4 DNA ligases at different concentrations (0.01, 0.02, 0.05, 0.08, 0.1, 0.2, 0.5, 0.8, 1.0, and 1.3 U/mL), the results of which are given in Figure 5A. We found that the fluorescence intensity increased with the concentration of the T4 DNA ligases. Additionally, there was still a certain degree of increment in fluorescence intensity that could be observed even at a low ligase concentration of 0.01 U/mL, which demonstrated that T4 DNA ligases could efficiently catalyze ligation reactions and induce a certain amount of fluorophore-containing hairpin structures at such a low concentration.

A regression analysis was performed with the fluorescence intensities collected at 561 nm, as shown in Figure 5B. According to the guideline EP17 [18], published by the Clinical and Laboratory Standards Institute (CLSI), the limit of detection (LoD) of our newly developed DNA probes on T4 DNA ligases is calculated as 0.055 U/mL as follows, which is at the same level as some of the previously reported susceptible methods [13,14].

Fit model: Logarithm (Log3P1)

Fitting equation: y = A − B × ln(x + C)

A = 34.70, B = −83.88, C = 0.89

Mean_blank_ = 0.00017 U/mL

SD_blank_ = 0.028 U/mL

SD_low concentration sample_ = 0.0053 U/mL

Limit of Blank: LoB was defined as the highest apparent analyte concentration expected to be found when replicates of a sample containing no analyte are tested. LoB is estimated by measuring replicates of a blank sample and calculating the mean result and the standard deviation (SD):

LoB = Mean_blank_ + 1.645 (SD_blank_) = 0.046 U/mL

Limit of Detection: LoD is determined by utilizing both the measured LoB and test replicates of a sample known to contain a low concentration of analyte. The mean and SD of the low-concentration sample are then calculated according to

LoD = LoB + 1.645 (SD_low concentration sample_) = 0.055 U/mL

## 4. Conclusions

In this study, we designed a highly sensitive and specific method for detecting DNA ligases, taking advantage of the unique properties of extraordinarily stable DNA mini-hairpins. The catalytic activity of DNA ligases was utilized to rejoin the nick sites that occurred within the nucleotide sequences of these hairpins, which stimulated the separation of the fluorophores and quenchers and further induced fluorescence signals from the DNA probes. It is the first time that the detection of DNA ligases with particular hairpin sequences has been achieved in a simple and fast manner, requiring only one reaction and mild conditions. This versatile detection method can be further applied to the real-time monitoring of intracellular DNA ligase activity, offering valuable insights for biomedical research. Currently, the insufficient cellular affinity of this biosensor prevents it from effectively entering cells. Our next steps involve transforming the dual-molecular probes into single-molecule ones and further integrating our fundamental principles with a variety of nanocarriers (e.g., quantum dots, liposomes, and polymeric micelles), which could help the probes extravasate into cells and tissues via enhanced permeability and retention effect. In addition, the native deoxyribonucleic acid backbones of the non-extraordinarily stable hairpin structure can be replaced with methyl- or phosphorothioate-modified backbones to improve its metabolic stability from nuclease-mediated degradation in serum. As research on cellular detection techniques advances, this approach could potentially evolve into diagnostic tools for various diseases and target identification in drug discovery in the future.

## Figures and Tables

**Table 1:** Examples of extraordinarily stable hairpins (Hairpin 1 and Hairpin 2) and ordinary DNA hairpins (Hairpin 3) [17].

Name	Structure	Stem Length	Stem GC Content	T_m_ (°C)
Hairpin 1 (extraordinarily stable hairpin used in our biosensor)	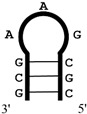	3 bp	100%	88.5
Hairpin 2 (extraordinarily stable hairpin)	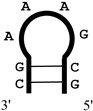	2 bp	100%	76.5
Hairpin 3 (ordinary hairpin)	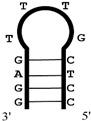	4 bp	75%	44.2

**Table 2:** Nucleotide sequences of the oligonucleotides used to construct DNA probes.

Name	Sequence (5′ to 3′)	Modification
Oligo 1	AAACTCCACGC	
Oligo 2	CTTCGCGTGGAGTTT	
Oligo 3	GAAGCGTGGAGTTTA	5′ P, 3′ Cy3
Oligo 4	TAAACTCCACG	5′ BHQ2

## Data Availability

The data presented in this study are available on request from the corresponding author.
